# Shabyar Ameliorates High Glucose Induced Retinal Pigment Epithelium Injury Through Suppressing Aldose Reductase and AMPK/mTOR/ULK1 Autophagy Pathway

**DOI:** 10.3389/fphar.2022.852945

**Published:** 2022-05-10

**Authors:** Xiao Yan Liu, Jun Peng, Fei He, Xirali Tursun, Shu Ping Li, Xue Lei Xin, Haji Akber Aisa

**Affiliations:** ^1^ The State Key Laboratory Basis Xinjiang Indigenous Medicinal Plant Resource, Xinjiang Technical Institute of Physics and Chemistry, Chinese Academy of Sciences, Urumqi, China; ^2^ University of Chinese Academy of Sciences, Beijing, China; ^3^ Xinjiang Institute of Materia Medica, Urumqi, China; ^4^ Institute of Xinjiang Traditional Uyghur Medicine, Urumqi, China

**Keywords:** shabyar, retinal pigment epithelium, autophagy, aldose reductase, traditional medicine formula

## Abstract

Shabyar (SBA) is a traditional medicine formula for relieving vision loss caused by factors including diabetic retinopathy (DR) in clinics. However, the mechanism of it on retina protective effect still unclear. The present study aimed to investigate whether its protective effect was related to aldose reductase (AR) inhibition and retinal pigment epithelial cell injury mediated by autophagy or not. Human retinal pigment epithelial cells (ARPE-19) induced by high glucose was used as a model *in vitro*, with Epalrestat (EPL, AR inhibitor) and Difrarel (DFR, DR therapeutic drug) as positive controls. Western blotting and Polyol pathway products assay showed that SBA reduced the expression of AR protein and the content of ROS, and sorbitol, increased the level of Na^+^-K^+^-ATPase and alleviated cell edema. Western blotting and DCFH-DA probe assay showed that SBA decreased pAMPK/AMPK and pULK1/ULK1 which associated with autophagy initiation, down-regulated Beclin-1, Atg3, Atg5, Atg7, LC3 II and Bax/Bcl2 ratio, and up-regulated pmTOR/mTOR, SQSTM1/p62 and mitochondrial membrane potential (MMP), reduces intracellular autophagosomes. Real-Time PCR assay showed that SBA had no significant effect on mRNA expression of AR and mTOR. These data demonstrated that SBA treatment inhibits the autophagy of ARPE-19 through the AMPK/mTOR/ULK1 signaling pathway, and reduced early-stage apoptosis occurred by high glucose. These findings reveal the protective role and mechanism of SBA on retinal pigment epithelium, and provide experimental basis for the clinical application of SBA in the treatment of DR.

## Introduction

Diabetic retinopathy (DR) is one of the most common microvascular complications of diabetes mellitus and a leading cause of blindness in working-age adults in developed countries (Liew et al., 2014). According to the severity of retinopathy, DR is classified as mild, moderate or severe non-proliferative DR (NPDR), proliferative DR (PDR) and diabetic macular edema (DME) and/or clinically significant macular edema (CSME). Glucotoxicity is involved in vascular damage through different metabolic pathways, such as production of advanced glycation end-products, activation of protein kinase C, polyol pathway activation and production of reactive oxygen species (Maranta et al., 2021). Among them the polyol pathway is activated, glucose is converted to sorbitol by aldose reductase (AR). Sorbitol cannot easily penetrate cellular membrane, accumulated in cells, leading to an increase in osmotic pressure, edema rupture, and permeability damage (Wu et al., 2018). Compared with nondiabetic patients, AR was highly expressed in the retinal pigment epithelial cells (RPE) of both eyes in more than 80% of PDR patients. Immunohistochemical results showed that the most intense staining of AR was occurred in the morphological damage area of RPE (Vinores et al., 1988). The outer blood-retina barrier (oBRB) is composed of RPE cell layer. As a permeable barrier between blood and retina, it has the function of maintaining retinal homeostasis (Yang et al., 2020). The thickness of the RPE layer is an optical coherence tomography (OCT) biomarker which is able to predict the functioning of the oBRB. DME-PDR patients showed thinner RPE layers in almost all quadrants, which was significantly different from that of DME-NPDR (Damian and Nicoara, 2020). Therefore, reducing the expression of AR in RPE and maintaining its morphological integrity might be a promising therapeutic strategy to alleviate the course of DR.

Autophagy and apoptosis pathways are crucial to prevent the progression of DR. Initially, autophagy is triggered in the dendrites of retinal ganglion cells through lysosomal metabolism of damaged organelles and denatured proteins, and cell was protected by self-renewal. When excessive autophagy is activated in the cytoplasm, cell death will be caused (Rosa et al., 2016). Activated AMPK promotes autophagy directly by phosphorylating autophagy-related proteins in the mTORC1, ULK1, and PIK3C3/VPS34 complexes (Li and Chen, 2019). High glucose treatment affects autophagy in human acute retinal pigment epithelial Cells (ARPE-19), and different results have been reported on whether autophagy protects or damages cells, which may be related to the time of glucose induction.

Shabyar (SBA) is a traditional medicine, which was recorded as a hospital preparation for hypopsia in the preparation standards of Chinese medical institutions (ISBN: 9787537254472) since 2013. Clinical double-blind, randomized, placebo-controlled trials showed that consumption of timolol plus SBA instead consuming of timolol alone was probably more effective for reducing intraocular pressure in patients with open-angle glaucoma (Khalil-BaniHabib et al., 2020). The result of our previous studies showed that SBA had AR inhibitory activity *in vitro*, IC50 was 61.79 μg/ml (Aisa et al., 2020). This suggests that the effect of SBA on reducing visual impairment may be related to the inhibition of AR. Therefore, the purpose of this study was to explore if the protective effect of SBA on ARPE-19 induced by high glucose is related to AR inhibition and autophagy regulation effect of it.

## Material and Methods

### Reagents and Instruments

In this study, the following reagents were used: Acetonitrile (Merck KGaA, Germany); formic acid (Sigma-Aldrich, United States); Difrarel (positive control for *in vitro* experiments, Laboratoires Leurquin Mediolanum SA, France), Epalrestat (positive control for *in vitro* experiments, Shanghai Yuan Ye Bio-Technology, China); DMSO (Vetec, United States); DMEM/F12 (11,320, Gibco, United States); FBS (12003C, Gibco, United States); DMEM (11,966, Gibco, United States); F12 (11,765, Gibco, United States); Penicillin-Streptomycin liquid (15,140, HyClone, United States); D-(+)-glucose (G7021, Sigma, United States); MTT (ThermoFisher Scientific, United States); D-sorbitol assay kit (Megazyme, Ireland); Na^+^-K^+^-ATPase detection kit (Solarbio Science and technology, China); DCFH-DA (Nanjing Jiancheng Bio-Engineering Institute, China); JC-10 MMP assay kit (Solarbio Science and Technology, China); RIPA lysis buffer (BOSTER, United States); BCA protein assay kit (A53225, ThermoFisher Scientific, United States); Trizol (Tianjin, China); Fastquant RT Kit (Tianjin, China); SYBR Green PCR kit (QIAGEN, Germany). Aloin (No. 110787), gallic acid (NO. 110831) and ellagic acid (NO. 111959) were obtained from the Chinese Food and Drug Accreditation Institute. All the primers were purchased from the Beijing Genomics institution (BGI, Shenzhen, China).

The following instruments were employed: Ultra performance liquid chromatography and Mass spectrometer (Q Exactive Focus, Thermo Fisher Scientific, United States); CO_2_ incubator (Binder, Germany); Electronic balance (LA120S, Sartorius, Germany); Ultrasound equipment (Xinyi, China); High-speed benchtop refrigerated centrifuge (Beckman, United States); M5 Multiplate reader (Molecular Device, United States); Nucleic acid protein extraction detection system (NanoDrop 2000C, ThermoFisher Scientific, United States); Real-time PCR system (7300 HT, Applied Biosystems, United States); Western ChemiDoc XRS imaging system (Bio-Rad, United States); Microtome (Leica, Germany); Inverted phase contrast microscope (DMi8, Leica, Germany); Transmission electron microscope (TEM 1230 (V), JEOL, Japan).

### Preparation of Shabyar and Positive Controls

SBA is a classic prescription composed of seven kinds of traditional Chinese medicine (Table 1). The ingredients were purchased from Xinjiang Xinlvbao Pharmaceutical Co., Ltd. and Xinjiang Maidisen Pharmaceutical Co., Ltd. All botanical drugs were identified by Associate Research Fellow Chunfang Lu from Xinjiang Technical Institute of Physics and Chemistry, Chinese Academy of Sciences. Voucher specimens (Barcode: *Aloe vera* gel (*Aloe vera* (L.) Burm. f. [*Asphodelaceae*]) WY02669, *Flos rosae rugosae* (*Rosa rugosa* Thunb. [*Rosaceae*]) WY02666, Trivrit (*Operculina turpethum* (L.) Silva Manso [*Convolvulaceae*]) WY02664, Mastix (*Pistacia lentiscus* L. [*Anacardiaceae*]) WY02665, *Fructus chebulae* (*Terminalia chebula* Retz. [*Combretaceae*]) WY02670, *Fructus foeniculi* (*Foeniculum vulgare* Mill. [*Apiaceae*]) WY02668, *Fructus tribuli* (*Tribulus terrestris* L. [*Zygophyllaceae*]) WY02667) were deposited at the Herbarium, Xinjiang Institute of Ecology and Geography, Chinese Academy of Sciences (XJBI). The preparation method of SBA extract was as follow: 1) A total of 103 g of the 7 botanical drugs was weighed. 2) 17 g *Flos rosae rugosae*, 15 g *Fructus chebulae*, and 14 g *Fructus tribuli* were decocted with deionized water (12 times the weight of botanical drug) for 2 h, and the extracting solution was collected. 3) Repeat the second step once and combine the extracting solution of the second and third steps. 4) Deionized water (14 times the weight of botanical drug) was heated to generate steam at 100°C to extract the volatile oil from 9 g *Fructus foeniculi* for 4 h, and the volatile oil and extracting solution were collected respectively. 5) The inclusion complex of *β*-cyclodextrin and *Fructus foeniculi* volatile oil was prepared, dried and crushed to obtain powder. 6) The extracting solution in steps 2, 3, and 4 were mixed, dried and crushed to obtain powder. 7) 30 g *Aloe vera* gel, 9 g Mastix, and 9 g Trivrit were crushed to obtain powder. 8) The SBA extract was obtained by mixing the three powders obtained in steps 5, 6, and 7. Details of the preparation method of SBA were showed in Supplementary Figure S1 and our patent (Aisa et al., 2020). The weight of dried extract from SBA accounts for 62% of the total weight of botanical drugs. According to our previously reported study, the characteristic components of Shabyar were quantitatively analyzed by Ultra-high-performance liquid chromatography with quadrupole- orbitrap high-resolution mass spectrometry to ensure the quality control of botanical drugs (Yan et al., 2022).

**TABLE 1:** SBA composition.

Botanical drug	Produced from	Dosage (g)
*Aloe vera* (L.) Burm.f. [*Asphodelaceae*; *Aloe vera* gel]	Dried matter of leaf fluid	30
*Rosa rugosa* Thunb. [*Rosaceae*; *Flos rosae rugosae*]	Dry flower petals	17
*Operculina turpethum* (L.) Silva Manso [*Convolvulaceae*; Trivrit]	Dry root	9
*Pistacia lentiscus* L. [*Anacardiaceae*; Mastix]	Resin	9
*Terminalia chebula* Retz. [*Combretaceae*; *Fructus chebulae*]	Fruit	15
*Foeniculum vulgare* Mill. [*Apiaceae*; *Fructus foeniculi*]	Fruit	9
*Tribulus terrestris* L. [*Zygophyllaceae*; *Fructus tribuli*]	Fruit	14

SBA extract and Difrarel powder were dissolved in DMSO at the concentration of 100 mg/ml, respectively. Eplarestat was dissolved in DMSO at the concentration of 100 *μ*M. Vortex at room temperature (23°C) for 5 min. The solutions were transferred to centrifuge tubes and stored at −20°C. It was mix with the medium and the mixture was filtered through a 0.22 *μ*m micropore membrane before use.

### UPLC-DAD-MS Analysis and Quality Control of Shabyar

SBA extract 100 mg was dissolved in 10 ml 50% methanol solution. The sample was sonicated for 15 min with an ice-water bath and centrifuged at 12,000 rpm for 10 min at 4°C, and filtered through a 0.45 *μ*m micropore membrane prior to injection. Finally, the supernatant was obtained and put in a fresh 2 ml brown tube for UPLC-MS analysis and main compounds content determination.

UPLC-DAD-MS analysis was performed on an Ultra-high performance liquid chromatography vanquish system (Q Exactive Focus, Thermo Fisher Scientific, United States), DAD (diode array detector), and G1311A quaternary solvent delivery system (Agilent, United States) with a Thermo RP C18 column (250 mm × 4.6 mm, id, ThermoFisher Scientific, United States). The composition of the mobile phase is C. Equate = “acetonitrile”, D. Equate = “0.3% HCOOH-H_2_O”. (See chromatographic conditions in Supplementary Table S1). 35°C column temperature. The DAD wavelength was set at an acquisition range of 200–600 nm, with an injection volume of 10 *μ*l and a flow rate of 1.0 ml/min.

The scan acquisition range was *m/z* 100–1,500 Da at 70,000 (FWHM), with a resolving power of *m/z* 200. The positive mode was used to obtain a spectral speed of 3 Hz with a HESI (heated electrospray) ion source. The following were the mass spectrometric parameters: 300°C as the heater temperature; 3.8 kV as the electrospray voltage; 350°C as the capillary temperature; nitrogen (N2) as the auxiliary and sheath gas; and helium (He) as the collision gas. The flow rate of the auxiliary gas and the sheath gas pressure were ten arb and 30 psi (1arb = 0.3 L/min), respectively. The collision energy (CE) was set between 30 and 70 eV.

An Orbitrap Q-Exactive high-performance benchtop MS analyzer system (Thermo Fisher Scientific, Germany) combined with an ultrahigh-pressure liquid chromatography (UHPLC) system (Thermo Fisher Scientific, Germany) was used for analysis. Analyst QS 2.0 software was used to perform data collection and processing.

### Cell Culture and Cell Viability Test

ARPE-19 cell were purchased from American Type Culture Collection (ATCC, United States). The cells were cultured in DMEM/F12 (v: v = 1:1) supplemented with 10% FBS, 100 U/ml penicillin and 100 *μ*g/ml streptomycin and incubated at 37°C with 5% carbon dioxide.

Suspended cells were seeded into 96-well plates at a density of 5×10^3^ cells/well. The cells were cultured in 4.5 mM or 30 mM glucose medium for 24 h, and then SBA with a final concentration of 0–200 *μ*g/ml was added for another 24 h. After treatment, the medium was removed, cells were washed with PBS, and 100 *μ*l MTT-containing medium (0.05%) was added to each well. After incubation for 4 h, cells were washed with PBS, the MTT-containing medium was replaced with 150 *μ*l/well DMSO to dissolve the formazan product formed by viable cells. The 570 nm OD value of each well was measured by microplate reader (Molecular Device, United States).

### Group Processing of Cell Experiments

The ARPE-19 were divided into seven groups: 1) Control group (glucose dose 4.5 mM); 2) Model group (glucose dose 30 mM); 3) Positive control one Difrarel group (glucose dose 30 mM and Difrarel 25 *μ*g/ml); 4) Positive control two Epalrestat group (glucose dose 30 mM and Epalrestat 0.1 *μ*M). (e-g) SBA group (glucose dose 30 mM and SBA 6.25–25 *μ*g/ml). The control or model group were treated in medium with 4.5 mM or 30 mM glucose for 48 h. The intervention group cells were treated in medium with 30 mM glucose for 24 h, then different concentrations of SBA or positive controls were added for other 24 h.

### Sorbitol Assay

Suspended cells were seeded into 6-well plates at a density of 3.5×10^5^ cells/well. After treatment of cells, the medium was removed and the cells were washed twice with cold PBS. Then the cells were collected into 120 *μ*l/well ultrapure water by a scraper. The collected cell was heated at 100°C for 10 min and centrifuged (8,000 g, 10 min) to remove the protein. The reagents were added to the supernatant according to the instructions of D-sorbitol assay kit (Megazyme, Ireland), and the absorbance was measured at 492 nm. Calculate the sorbitol concentration according to the formula provided in the kit operation manual.

### Na^+^-K^+^-ATPase Assay

After treatment of cells, the medium in 6-well plate was removed and the cells were washed twice with cold PBS, then 200 *μ*l 0.25% Trypsin-EDTA (Gibco, United States) was added 2 min later, Trypsin was removed and reagent one in Na^+^-K^+^-ATPase kit’s (Solarbio Science and technology, China) was added. The cell concentration was adjusted to 5×10^6^/ml and ultrasonic crushing was performed at 4°C. After centrifugation, the supernatant was taken, reagents were added according to the operation manual of Na^+^-K^+^-ATPase detection kit, and absorbance was measured at 660 nm.

### Reactive Oxygen Species Assay

After treatment of cells, 1.5 ml/well DCFH-DA (Nanjing Jiancheng Bio-Engineering Institute, China) was added in 6-well plate and incubated for 1 h in the dark. The medium was removed and the cells were washed with cold PBS and 200 *μ*l 0.25% Trypsin-EDTA (Gibco, United States) was added. After Trypsin was removed and 320 *μ*l of PBS was added. The cell concentration was adjusted to 1×10^7^/ml. 100 *μ*l/well cell suspension was added to fluorescent plate, and the fluorescence data were read at the excitation wavelength of 485 nm and the emission wavelength of 525 nm. The above assays were tested three times for each sample.

### Mitochondrial Membrane Potential Assay

According to the kit instructions, referring to the method in reference (Shivarudrappa and Ponesakki, 2020), the JC-10 MMP assay kit (Solarbio Science and Technology, China) was used to detect changes in MMP in each group. Each sample was analysed according to three independent wells. Dmi8 inverted phase contrast microscope (Leica, Germany) was used for observation and photographing. The results were read and analysed by Image J software.

### Transmission Electron Microscopy

Cells were collected in FBS and were fixed with glutaraldehyde at 4°C. Then the samples were fixed with 1% osmium tetroxide for 1 h and washed with 0.07 M phosphate buffer. Gradient dehydration was performed with different concentrations of acetone. The samples were embedded in araldite and 50 nm ultrathin section was prepared and was stained with 3% uranium acetate and lead citrate. The prepared sample slides were scanned by transmission electron microscopy [TEM 1230 (V), JEOL, Japan].

### Western Blotting Analysis

The total protein of cells was extracted by RIPA buffer with phosphatase inhibitor, and the concentration of total proteins was measured by BCA kit (Thermo Scientific, United States). Equivalent amounts of protein (35 *μ*g) of each sample were subjected to 8% SDS-PAGE, and then transferred onto a polyvinylidene difluoride (PVDF) membrane. The PVDF membrane was blocked with 5% skimmed milk for 1 h. Subsequently, the membrane was incubated with primary antibody (1:1,000) at 4°C overnight. After washed in TBST for three times, the membrane was blotted with secondary antibody (1:5000) at room temperature for 1 h and the specific bands were detected with the enhanced chemiluminescent reagent (ECL, Sigma, Unitd States). The housekeeping gene (GAPDH) or *β*-actin was used as an endogenous reference. Antibody and supplier information were presented in Table 2. ChemiDoc XRS imaging system (Bio-Rad, United States) was used to detect the enhanced chemiluminescence of the protein bands. Results were analysed by Adobe Photoshop CC 2017. Each sample was analysed according to three independent blots.

**TABLE 2:** Antibody and Supplier Information.

Vendor	Primary antibodies	Product NO.	MW
abcom	Anti-Aldose reductase Antibody	ab179354	35kd
CST	Phospho-AMPKα (Thr172) (40H9) Rabbit mAb	2,535	62Kd
CST	AMPKα (D5A2) Rabbit mAb	5,831	62kd
CST	Phospho-mTOR (Ser2448) Antibody	2,971	289Kd
CST	mTOR Antibody	2,971	290Kd
CST	Phospho-ULK1 (Ser555) (D1H4) Rabbit mAb	5,869	140-150Kd
CST	ULK1 (D8H5) Rabbit mAb	8,054	150kd
CST	Beclin-1 (D40C5) Rabbit mAb	3,495	60kd
CST	Atg3 Antibody	3,415	40kd
CST	Atg5 (D5F5U) Rabbit mAb	12,994	55kd
CST	Atg7 (D12B11) Rabbit mAb	8,558	78kd
CST	LC3A/B (D3U4C) Rabbit mAb	12,741	14, 16kd
CST	SQSTM1/p62 Antibody	5,114	62Kd
CST	Bax (D2E11) Rabbit mAb	5,023	20Kd
CST	β-Actin (13E5) Rabbit mAb	4,970	45kd
CST	GAPDH (D16H11) Rabbit mAb	5,174	37kd
CST	β-tubulin antibody	2,146	55kd
absin	Rabbit anti-Bcl2 Polyclonal Antibody	ABS131701	26-30Kd
absin	Goat anti-rabbit IgG-HRP	ABS20040	

### Real-Time PCR

Total RNA was extracted using Trizol (Tianjin, China) reagent based on the manufacturer’s protocols. Total RNA was reverse transcribed to synthesize cDNA by fastquant RT Kit (Tianjin, China). cDNA was amplified using SYBR Green PCR kit (QIAGEN, Germany) and 7300 HT Real-time PCR system (Applied Biosystems, United States). Real-Time PCR detection was based on the method published by Liu et al. (2018). The primers are presented in Table 3. The housekeeping gene (GAPDH) was used as internal control. The mean cycle threshold (Ct) values of the gene were determined using the two ^ΔΔ^Ct method. Each sample was tested three times.

**TABLE 3:** Primer sequences.

Target gene	Nucleotide sequences (5 to 3)
	Upper strand: Sense
	Lower strand: Antisense
AR	5′-TTT​TCC​CAT​TGG​ATG​AGT​CGG-3′
	5′-ACG​TGT​CCA​GAA​TGT​TGG​TGT-3′
GAPDH	5′-GTC​TCC​TCT​GAC​TTC​AAC​AGC​G-3′
	5′-ACC​ACC​CTG​TTG​CTG​TAG​CCA​A-3′
mTOR	5′-GTG​GTG​GCA​GAT​GTG​CTT​AG-3′
	5′-TTC​AGA​GCC​ACA​AAC​AAG​GC-3′

### Statistical Analysis

The data were expressed as the mean ± standard deviation (SD). Differences among groups were analysed by using One-Way ANOVA and Donnet’s post-analysis or Tukey’s multiple comparison test, and *p* < 0.05 was considered significant. All analyses were performed using GraphPad Prism software 8 (GraphPad Software, United States).

## Results

### Chemical Profiling of Shabyar

16 known compounds were detected in SBA by UPLC-DAD-MS analysis (Table 4). The methods and results of quantitative analysis for three main components aloin, gallic acid and ellagic acid, were shown in the Supplementary Tables S2, S3.

**TABLE 4:** Compounds identified in SBA *via* UHPLC-DAD-Quadrupole-Orbitrap-MS.

No	Rt (min)	m/z	Proposed formula	Error (ppm)	MS2 data (m/z)	Identification
1	3.66	191.0556	C_7_H_12_O_6_	3.53	173, 160, 127, 109, 102, 93, 85, 71, 59	Quinic acid
2	7.46	169.01360	C_7_H_6_O_5_	2.66	125, 123, 107, 97, 79, 69	Gallic acid
3	14.36	633.07458^-^	C_27_H_22_O_18_	3.70	301, 275, 257, 229, 145	Galloyl-HHDP-glucoside
4	20.99	615.10016^-^	C_28_H_24_O_16_	3.41	435, 351, 300, 271, 255, 243, 229	Quercetin-galloyl-glucoside
5	21.09	409.11478	C_19_H_22_O_10_	4.54	247, 215, 203, 188, 171, 159, 127	Aloenin A
6	21.15	433.11450	C_21_H_22_O_10_	3.64	270, 253	Hydroxyaloin
7	22.87	609.14709	C_27_H_30_O_16_	3.41	300, 271, 255, 243, 151	Rutin
8	23.78	300.99936	C_14_H_6_O_8_	4.87		Ellagic acid
9	24.62	433.11475^-^	C_21_H_22_O_10_	4.22	270, 253	Hydroxyaloin
10	24.62	463.08911	C_21_H_20_O_12_	4.33	300, 271, 255, 243, 151	Quercetin glucoside
11	29.08	717.20471^-^	C_34_H_38_O_17_	3.04	555, 409, 307, 247, 203, 188, 171, 145, 123, 119	Aloenin B
12	33.61	247.06129	C_13_H_12_O_5_	4.82	215, 203, 188, 171, 159, 127, 123	Aloenin aglycone
13	35.76	417.12007	C_21_H_22_O_9_	4.99	297, 268, 251, 225	Aloin A/B
14	39.05	555.18756	C_29_H_32_O_11_	2.65	435, 393, 89, 247, 214, 160, 147	Aloeresin D
15	40.30	417.11957	C_21_H_22_O_9_	3.74	297, 268, 251, 225	Aloin A/B
16	48.79	563.17749	C_27_H_32_O_13_	2.79	443, 279, 251	Aloinoside B/A

### Conditional Screening of ARPE-19 Cell Model With High-Glucose Injury

**FIGURE 1:**
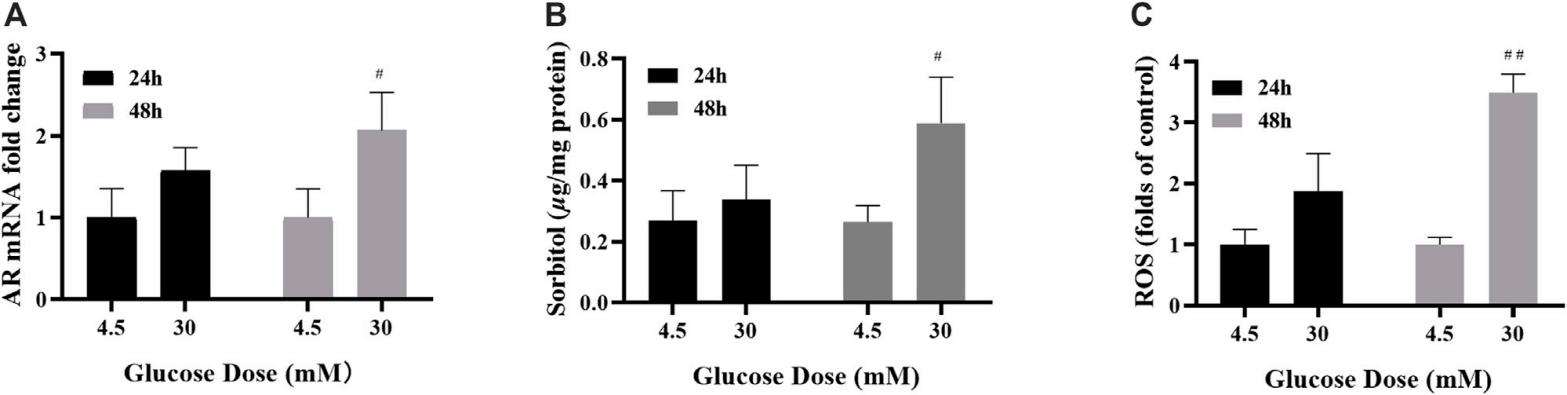
48 h of high-glucose stimulation was the appropriate modelling condition. **(A–C)** The levels of AR mRNA, sorbitol and ROS in ARPE-19 cells under different high glucose stimulation duration. *n* = 3 per group. #*p* < 0.05, ##*p* < 0.01 vs. glucose dose 4.5 mM.

In this study, cells were treated with control (4.5 mM glucose) or high-glucose (30 mM glucose) medium. The optimal time were determined by AR mRNA, ROS and sorbitol. The levels of AR mRNA, sorbitol and ROS were increased significantly in ARPE-19 treated with 30 mM glucose for 48 h (Figures 1A–C) and 48 h was determined as the optimal stimulus time.

### High Glucose Induced Autophagy in ARPE-19 Through AMPK Pathway

AMPK is one of the key activators of autophagy, which starts autophagy by phosphorylating Unc-51 like autophagy activating kinases (ULK1). In order to investigate the changes of autophagy in high glucose induced ARPE-19 cells, the ratio of pAMPK (Thr172)/AMPK induced by glucose for 1–72 h was detected.

**FIGURE 2:**
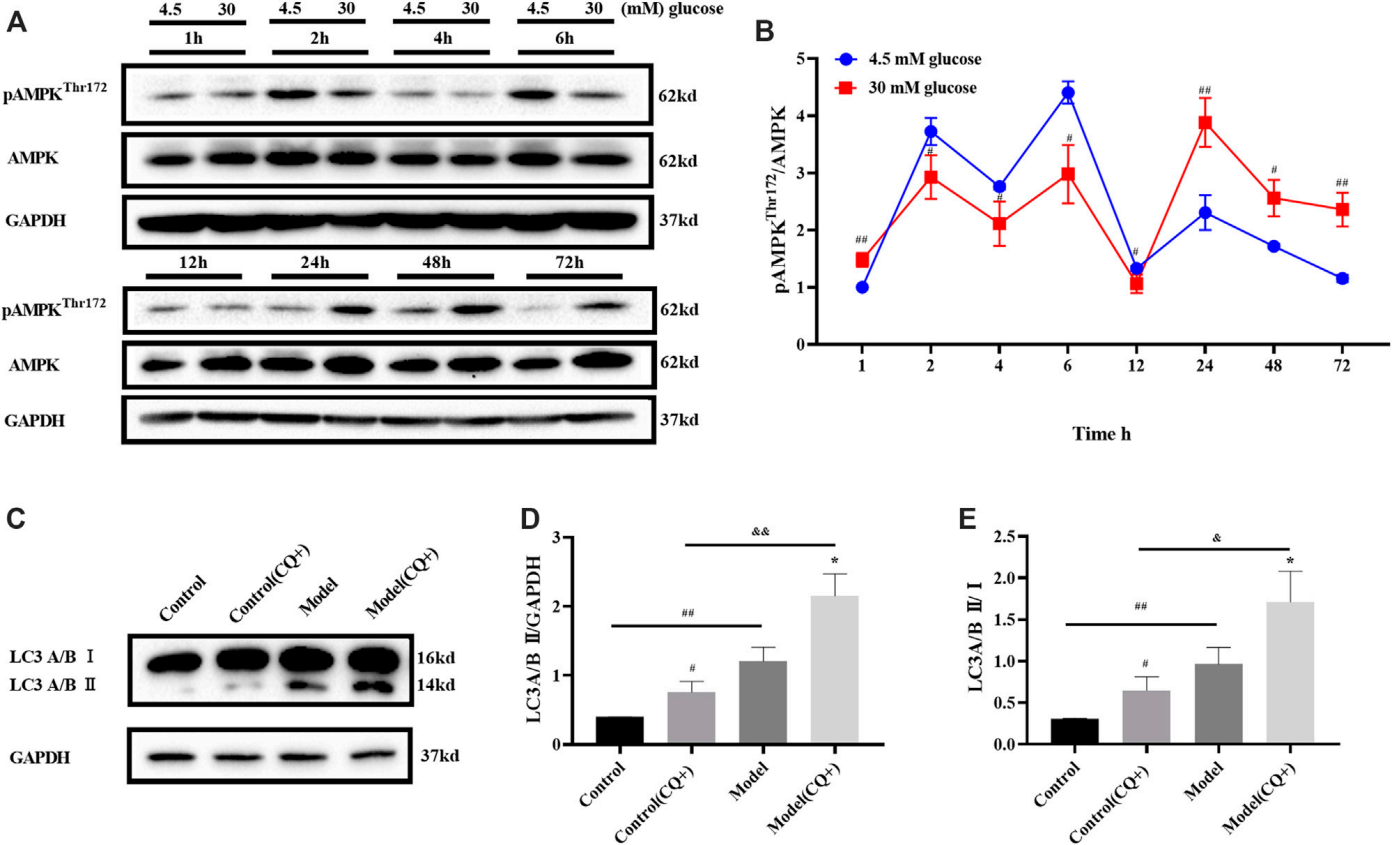
High glucose induced autophagy in ARPE-19 through AMPK pathway. **(A,B)** pAMPK/AMPK ratio changed with high glucose induction time. **(C–E)** LC turnover assay result. CQ: chloroquine. *n* = 3 per group. **(B,D)**: #*p* < 0.05, ##*p* < 0.01 vs. control; *p < 0.05, **p < 0.01 vs. model; && *p* < 0.01 vs. control (CQ+). **(E)**: *p < 0.05 vs. CQ-group.

The results of Western Blot showed that the phosphorylation state of AMPK fluctuated with time under the same glucose concentration. When the groups with different glucose concentrations were compared with each other at the same time point, the pAMPK/AMPK ratio of ARPE-19 treated with 30 mM glucose for 1 h was higher than the control group, and the ratios were reversed compared with the previous time after 2–12 and 24 h respectively (Figure 2A). The pAMPK/AMPK ratio in the model was higher than the control, until 72 h (Figure 2B). Therefore, when the polyol pathway was activated, the ratio of pAMPK/AMPK was increased.

The static level of LC3 II detected by Western blot could not fully reflect the changes of autophagy flow in cells. There are two possibilities when LC3 II increases: 1) more LC3 II was involved in the formation of autophagosomes when autophagy was activated; 2) LC3 II degradation was inhibited when the autophagy tide was inhibited at late stage. At this time, it could be distinguished by LC3 turnover assay in combination with lysosomal inhibitors. Herein, LC3 turnover assay was detected after 48 h of glucose induction.

Cells treated with chloroquine (CQ, an autophagy inhibitor, Lysosomal inhibitors) could not undergo lysosomal digestion, the degradation of LC3 II was inhibited, and autophagy was blocked in the cytoplasm (Mauthe et al., 2018). In the LC3 turnover assay, cells in the CQ + group were treated with 10 *μ*M CQ for 1 h before glucose treatment. LC3 turnover assay results showed that LC3 II in model group was higher than that in the control group when CQ did not intervene. Because CQ inhibited LC3 II degradation, LC3 II in the control group (CQ+) was higher than that in the control group. Meanwhile, LC3 II in model (CQ+) group was higher than that in the model group, that is, when CQ existed, the degradation of LC3 II was inhibited, and the LC3 II induced by high glucose further increased, ruling out the possibility that high glucose inhibited the autophagy flow at late stage and led to the increase of LC3II. LC3 II/Ⅰ ratio changes were consistent with LC3 II protein expression (Figures 2C–E). This suggests that high glucose can up-regulate autophagy and lead more LC3 II participate in autophagosome formation. The induction effect of high glucose on ARPE-19 was consistent with the result in the reference (Yoshii and Mizushima, 2017).

In conclusion, when ARPE-19 cells were treated with 30 mM glucose for 48 h, AMPK was activated, LC3 II expression was significantly increased, and autophagy was activated.

### Shabyar Inhibits Aldose Reductase Activation in ARPE-19 Induced by High Glucose

**FIGURE 3:**
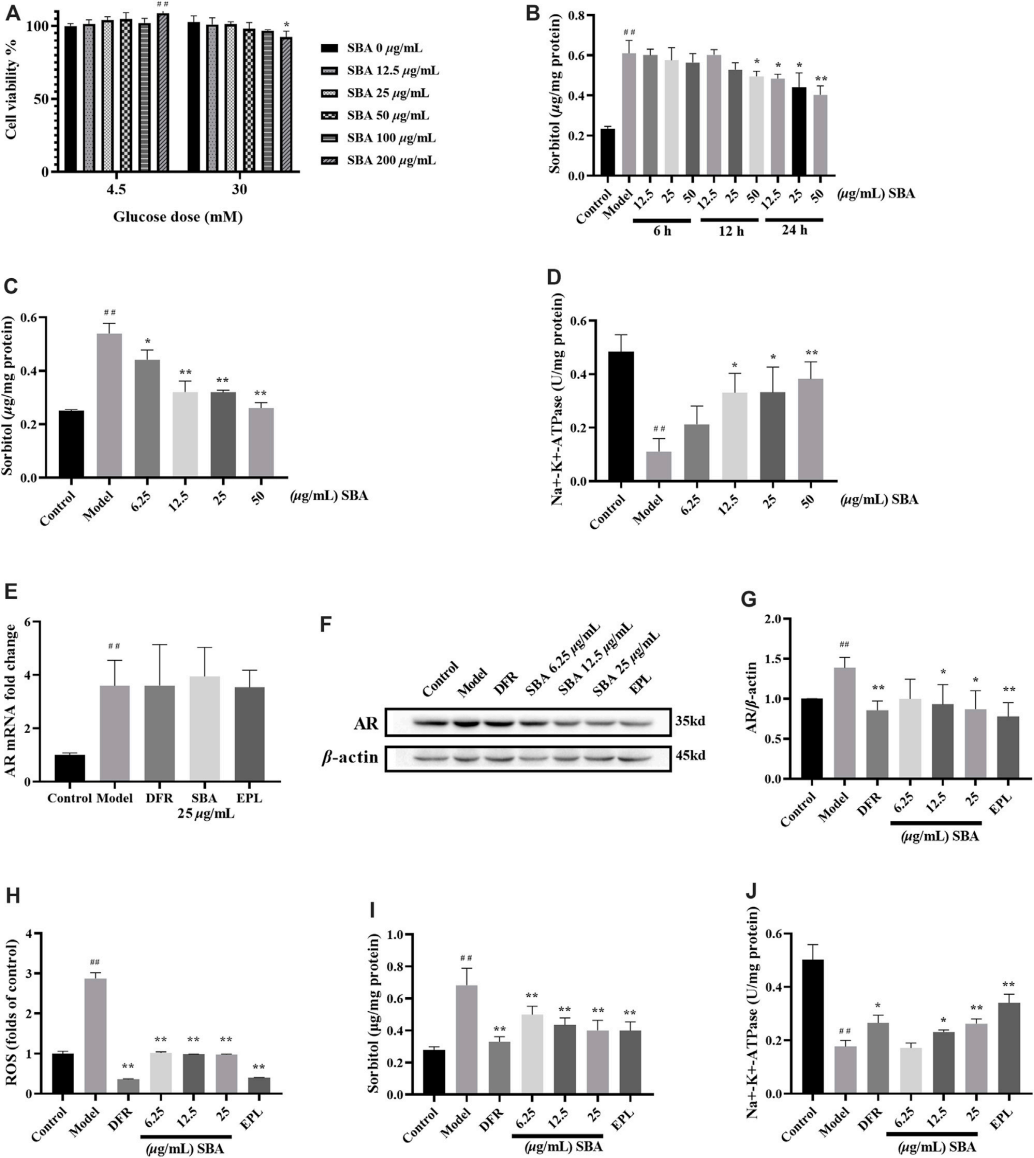
SBA reduced AR protein and downstream product levels in high glucose induced ARPE-19. **(A)** Effect of SBA on cell viability (glucose dose was 4.5 mM or 30 mM). **(B)** Effects of different SBA durations (6–24 h) on intracellular sorbitol levels when glucose stimulated fixation (30 mM, 48 h). **(C,D)** Effects of SBA acted on cells for 24 h on sorbitol and Na+-K+-ATPase. **(E)** AR mRNA was up-regulated in ARPE-19 induced by high glucose. mRNA levels were normalized to the control group values. **(F,G)** Effect of SBA on AR protein of ARPE-19 induced by high glucose. **(H–J)** ROS, sorbitol and Na+-K+-ATPase test results. **(A–H)**, **(J)**: *n* = 3 per group, **(I)**: *n* = 6 per group. **(A)**: ##p < 0.01 vs. glucose dose 4.5 mM + SBA 0 μg/ml, *p < 0.05 vs. glucose dose 30 mM + SBA 0 μg/ml. **(B–D)**: #p < 0.05, ##p < 0.01 vs. control, *p < 0.05, **p < 0.01 vs. model.

Cell viability results showed that, SBA (below 100 *μ*g/ml, 24 h) was safety for ARPE-19 no matter the glucose concentration of the medium was 4.5 mM or 30 mM (Figure 3A).

The optimal action time and dose of SBA were determined by sorbitol and Na^+^-K^+^-ATPase level. The intervention effect of SBA with different duration of action on model was observed within 48 h of exposure to high glucose. After treated with 30 mM glucose 42/36/24 h, SBA was added, and cells were treated with 30 mM glucose and SBA for another 6/12/24 h. The results showed that after treated with 12.5–50 *μ*g/ml SBA 24 h, the intracellular sorbitol level decreased in a dose-dependent manner (Figure 3B). The risk of clinical medication is related to the effective dose. Taking the activity of sorbitol and Na^+^-K^+^-ATPase as indicators, the lower effective concentration of SBA was screened. Under the same treatment conditions, the intracellular sorbitol level decreased and Na^+^-K^+^-ATPase increased in ARPE-19 cell model in a dose-dependent manner after treated with 6.25–50 *μ*g/ml SBA for 24 h (Figures 3C,D). Therefore, the optimal dose of SBA for 24 h was 6.25–25 *μ*g/ml.

SBA or positive controls could not reverse the increase of AR mRNA induced by high glucose (Figure 3E). Instead, SBA or positive controls significantly reversed the increase of AR protein, sorbitol, ROS levels and the decrease of Na^+^-K^+^-ATPase in ARPE-19 cells induced by high glucose (Figures 3F–J). These data showed that SBA could reversed the effects of high glucose induction on AR protein expression, sorbitol, ROS and Na^+^-K^+^-ATPase activities of ARPE-19. The similar results also be found in AR inhibitor EPL and DR therapeutic drug DFR which were used as positive control.

### Shabyar Decreased Autophagy Induced by High Glucose by Inhibiting AMPK/mTOR/ULK1 Pathway

**FIGURE 4:**
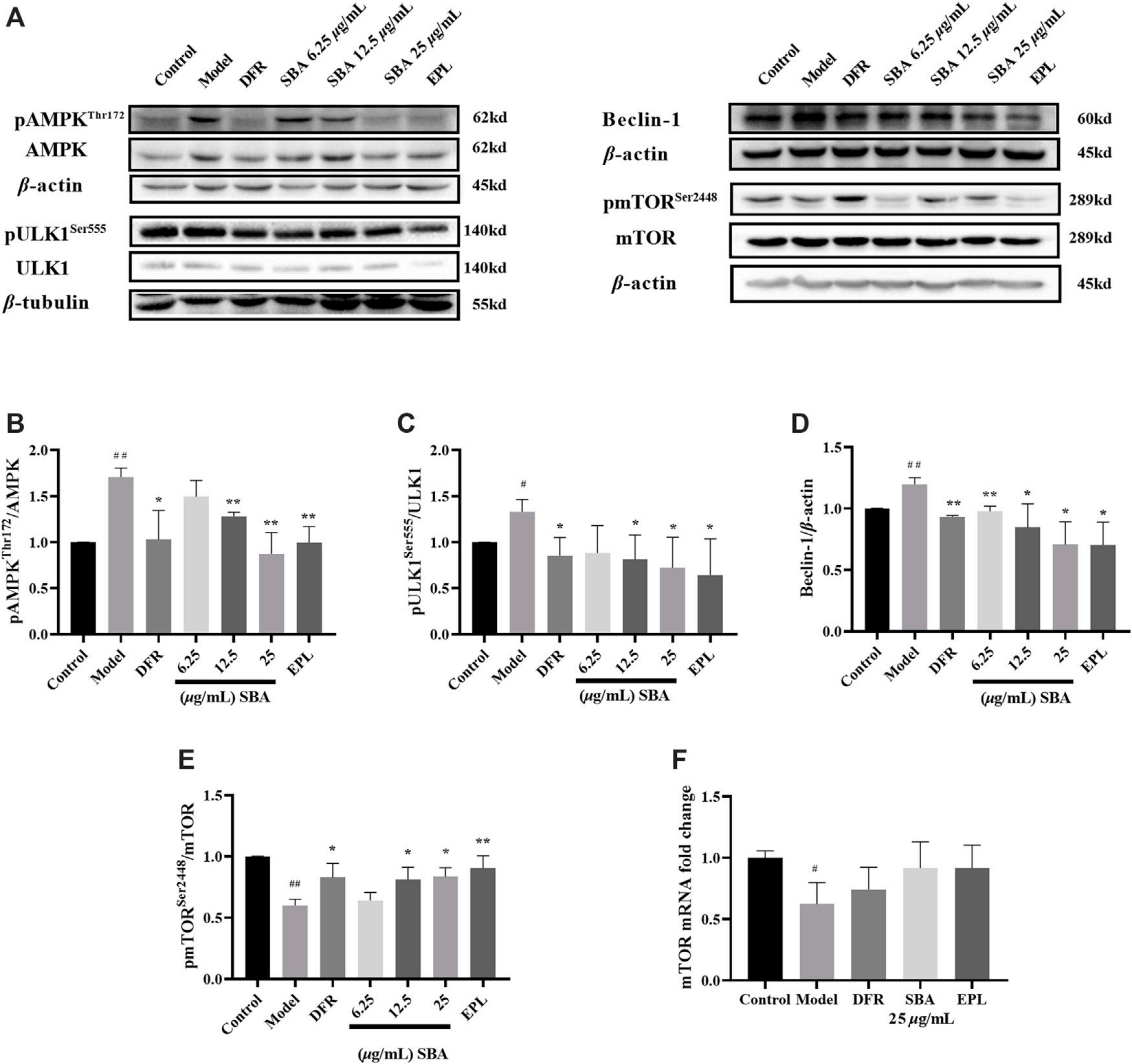
SBA inhibits autophagy through AMPK/mTOR/ULK1 pathway. **(A–E)** Western blot results of AMPK, ULK1, Beclin-1, and mTOR. **(F)** High glucose-induced the downregulation of mTOR mRNA level in ARPE-19. *n* = 3 per group. #p < 0.05, ##p < 0.01 vs. control, *p < 0.05, **p < 0.01 vs. model.

In this study, the expression level and mRNA level of key proteins associated with autophagy in AMPK/mTOR/ULK1 pathway were detected. The results showed that the key proteins like pAMPK/AMPK, pULK1/ULK1 ratio and Beclin-1 increase and pmTOR/mTOR decreased significantly in ARPE-19 induced by high glucose (Figure 4A), and mRNA expression of mTOR also decreased significantly. SBA and positive controls significantly reversed the expression of these proteins (Figures 4B–E). Meanwhile, mRNA expression of mTOR in SBA and positive control groups increased, but there was no significant difference compared with the model group (Figure 4F). These results suggest that SBA may affect autophagy through AMPK/mTOR/ULK1 pathway. EPL and DFR may also affect autophagy in the same way. This result is similar to that of Pal, they found that AR inhibitors Fidarestat regulate SIRT1/AMPK-*α*1/mTOR pathway in Human Umbilical Vein Endothelial Cells (HUVECs) induced by high glucose (Pal et al., 2019).

### Shabyar Inhibits Autophagy Induced by High Glucose

LC3 is the most widely used maker in autophagy. LC3 I combines with phosphatidyl ethanol (PE) to form LC3 II, which is located in the isolation membrane and autophagosome. The number of LC3 II reflects the number of autophagosomes and autophagy-related structures. The degradation of SQSTM1/p62 is another widely used marker to monitor autophagy. Because SQSTM1/p62 directly binds to LC3 and is selectively degraded by autophagy, the autophagy flux increases when LC3 is up-regulated and p62 is down-regulated (Yoshii and Mizushima, 2017).

**FIGURE 5:**
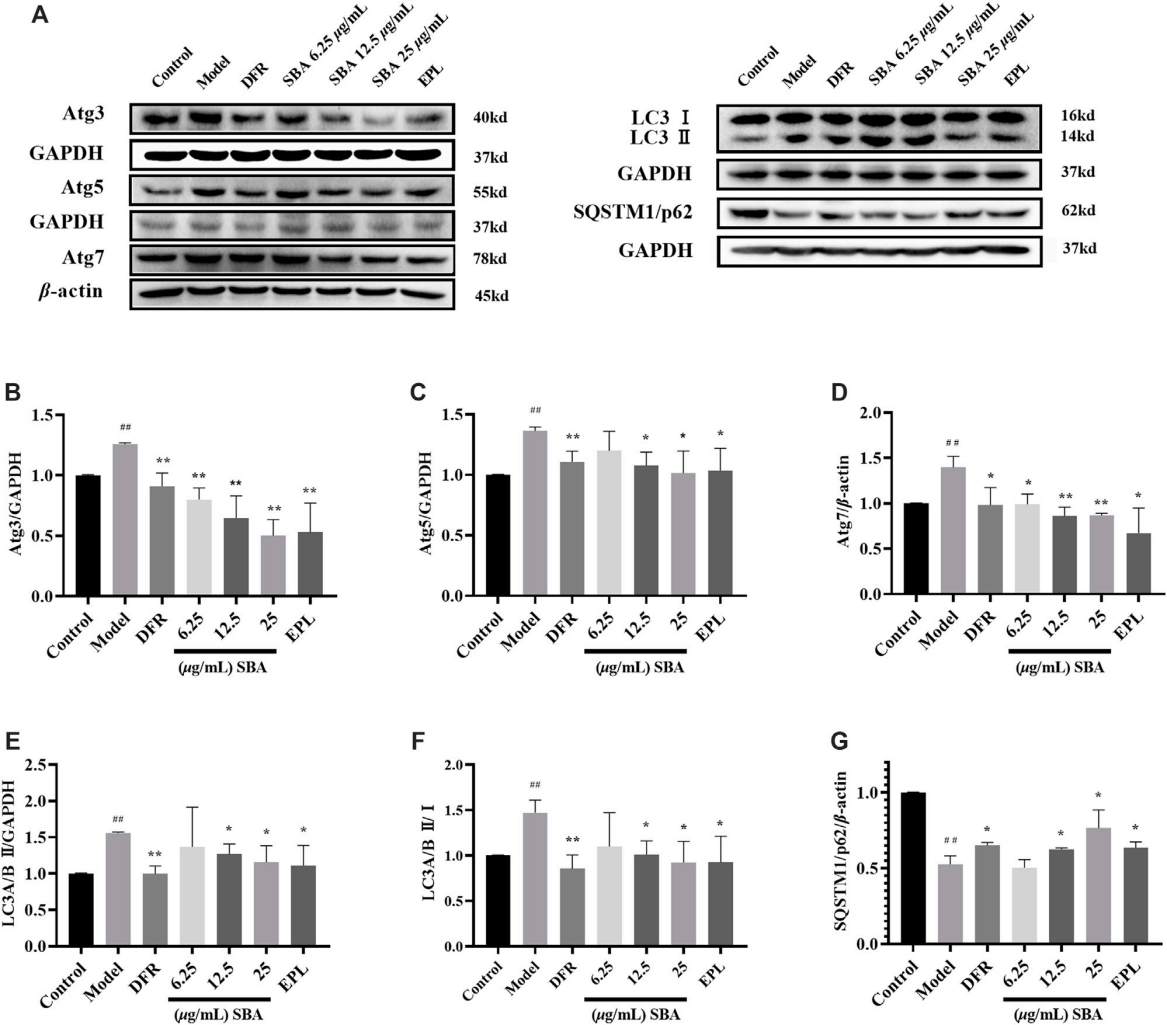
SBA inhibits high glucose induced autophagy in ARPE-19. **(A–G)** Western blot results of Atg3, Atg5, Atg7, LC3 II, and SQSTM1/p62. *n* = 3 per group. #p < 0.05, ##p < 0.01 vs. control, *p < 0.05, **p < 0.01 vs. model.

In this study, the expression of proteins related in autophagy such as LC3 II, SQSTM1/p62, Atg3, Atg5 and Atg7 were detected by WB assay, the results showed that all these proteins except SQSTM1/p62 were significantly elevated in high glucose induced ARPE-19 (Figure 5A). SBA and positive controls significantly reversed the expression levels of these proteins which induced by high glucose (Figures 5B–G). The data showed that autophagy was enhanced in ARPE-19 induced by high glucose, while SBA could regulate autophagy related proteins and inhibit autophagy, these results also be seen in positive control group. This may suggest that AR inhibitors have a regulatory function on autophagy.

### Shabyar Protects Cells From High Glucose Injury and Reduces the Number of Autophagosomes in ARPE-19 Cells

**FIGURE 6:**
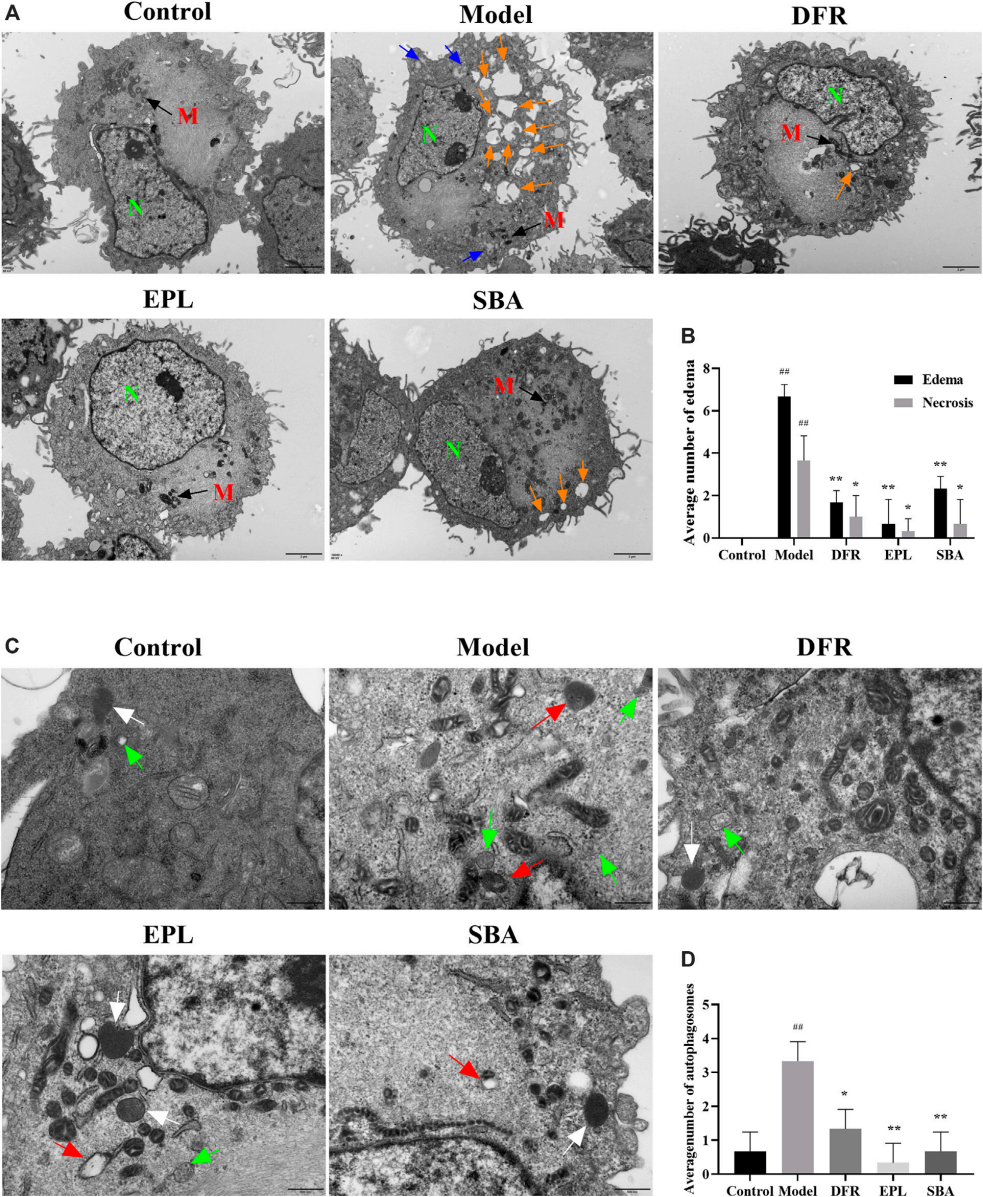
TEM observation results. **(A)** Scale bar: 2 μm. Orange arrow: edema; blue arrow: necrosis. N, nucleus; M, mitochondria. **(B)** Number of edema and necrosis in different groups is presented as bar graphs. **(C)** Scale bar: 500 nm. Green arrow: autophagosomes; white arrow: lysosomes; red arrow: autolysosome. **(D)** Quantification of the autophagosomes in different groups is presented as bar graphs. The figures are representative images of three different samples. SBA group dose 25 μg/ml *n* = 3 per group. #p < 0.05, ##p < 0.01 vs. control, *p < 0.05, **p < 0.01 vs. model.

In order to further determine the characteristics of ultrastructure and autophagy state in ARPE-19, TEM observation was carried out. TEM observation showed that edema (Round like vesicles with clear boundary, orange arrow in Figure 6A), necrosis (Gray area with clear boundary, blue arrow in Figure 6A) and obvious cell damage were widely distributed in the model group. SBA and positive controls reversed this situation, with significantly reduced intracellular edema and necrosis (Figure 6B). These results indicate that both SBA and positive control could reduce the damage of cell ultrastructure caused by high glucose.

Autolysosome are formed by the combination of autophagic vesicles with lysosomes or the phagocytosis of autophagic vesicles by lysosomes. The process in which their contents are degraded by lysosomal enzymes is autophagy. Lysosomes are common organelles in eukaryotic cells. Autolysosome are also widely present in normal cells. When autophagy is activated, the number of autolysosomes increase significantly in response to stimuli. The TEM results showed that the numbers of autophagosomes (double-or multiple-membrane vacuoles containing cytoplasmic material and organelles, green arrow in Figure 6C) and autolysosome (single-membrane structures with partially digested content, red arrow in Figure 6C) were significantly increased in ARPE-19 treated with high glucose (Figure 6D). Autophagosomes, lysosomes (a bubble-like structure surrounded by a monolayer film and the contents are uniform, white arrow in Figure 6C) and or autolysosome containing partially degraded cellular substances were seen in cell of SBA, positive control groups, and control group. Compared with the model group, autophagosomes in SBA group was significantly reduced, and so was the positive control. (Figure 6D). The results showed that both SBA and positive controls could reduce the number of autophagosomes in ARPE-19.

### Shabyar Decreased Early-Stage Apoptosis in High Glucose-Induced ARPE-19

**FIGURE 7:**
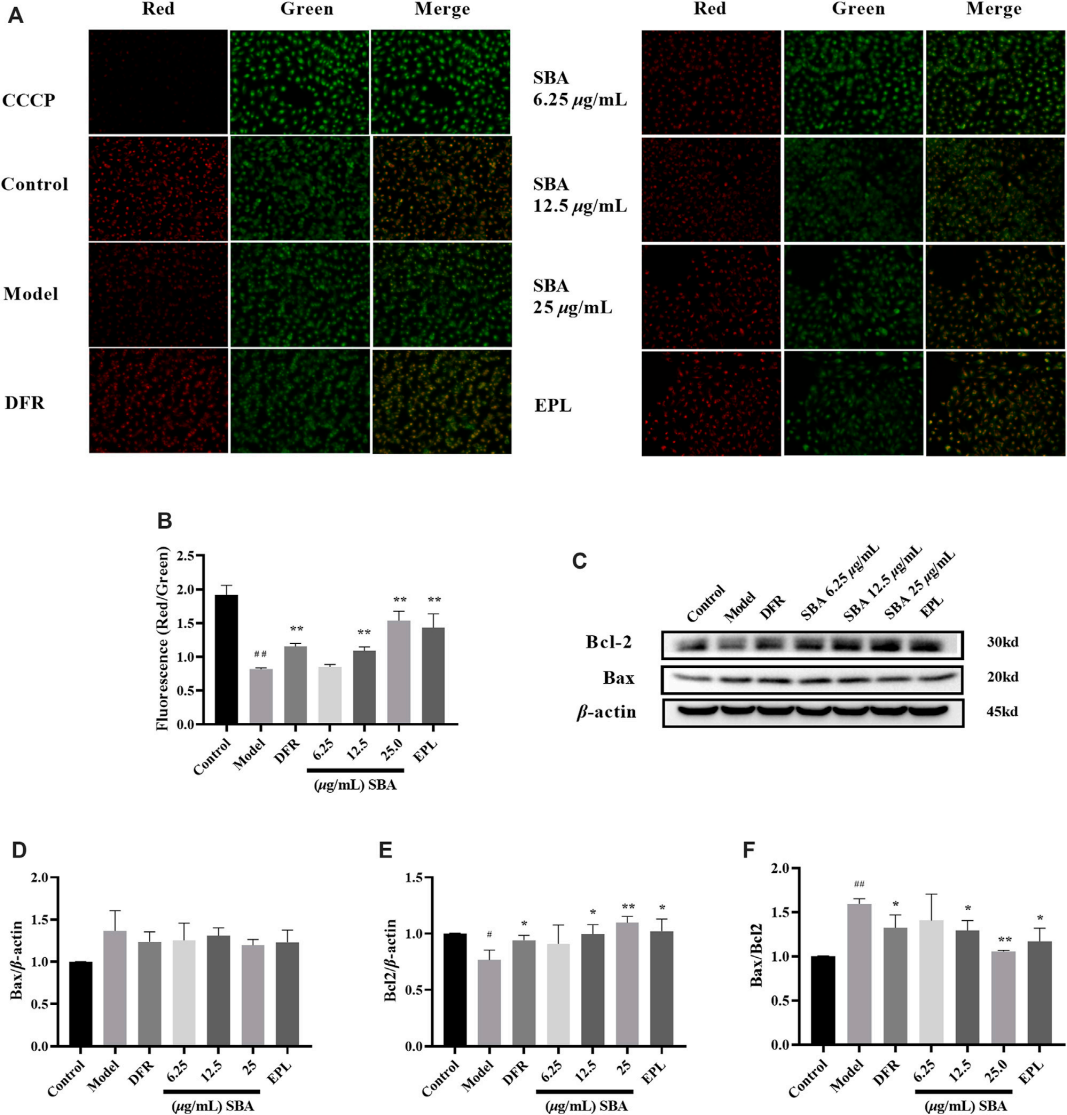
SBA decreased early-stage of apoptosis. **(A)** MMP fluorescence images of each group. **(B)** Quantitative analysis results of MMP. **(C)** Western blot results of Bax and Bcl2. **(D–F)** Bax, Bcl2, Bax/Bcl2 levels in ARPE-19. *n* = 3 per group. #p < 0.05, ##p < 0.01 vs. control, *p < 0.05, **p < 0.01 vs. model.

The decline in MMP is one sign of the early stages of apoptosis on cells. The change of MMP in the early stage of apoptosis induced changes on the permeability of membrane. The increase of membrane permeability will lead to releases more mitochondrial proteins such as Cytochrome C from the mitochondrial matrix to the cytoplasm. The release of Cytochrome C is accompanied by the complete loss of membrane potential, which leads to the cascade effect of apoptotic enzymes. The membrane potential in control group cells was normal, and JC-10 dye accumulated in mitochondria in a potential dependent manner to form a polymer and emit red fluorescence; in the cells with early-stage apoptosis, the membrane potential decreases or loses, JC-10 dye only exists in the cytoplasm in the form of monomer and emits green fluorescence. Apoptosis inducer carbonyl cyanide m-chlorophenyl hydrazone (CCCP) was used as an indicator. The control cells maintained a brighter red fluorescence intensity, while the red fluorescence intensity and MMP decreased in model group. SBA and positive controls could significantly restore the red fluorescence intensity (Figures 7A,B). Meanwhile, high glucose significantly reduced expression level of Bcl2 protein in ARPE-19 cells, resulting in a significant increase in Bax/Bcl2 ratio, and SBA and positive controls significantly reversed the above trends (Figures 7C–F). The results showed SBA could reduce the early stage of apoptosis in ARPE-19 after high glucose exposure, these results also be observed in EPL and DFR groups.

## Discussion

In this study, human retinal pigment epithelial ARPE-19 cells were used to construct a cell damage model by high-glucose stimulation. RPE is an important part of oBRB, and ARPE-19 has been used as accepted cellular models *in vitro* for retinal diseases such as DR and Age-related macular degeneration (AMD). In ARPE-19 cells, the induction of 30 mM glucose (48 h) activated autophagy, increased the number of autophagosomes, up-regulated ROS, and significantly increased the expression of Bcl2/adenovirus E1B 19 kDa interacting protein 3-like (BNIP3L) and PTEN induced putative kinase 1 (PINK1), autophagy disorder mediates damage of retinal pigment epithelial cells by hyperglycemia (Huang et al., 2018; Zhang et al., 2021). In diabetic patients, the damaged RPE expressed a higher level of AR (Vinores et al., 1988). In hyperglycaemia, glucose is metabolized to sorbitol *via* AR, which reduces NADPH levels and causes intracellular oxidative imbalance and ROS accumulation (Kang and Yang, 2020). Sorbitol accumulates in cells, form osmotic gradient, which damages cell structure and function, hinders inositol uptake, and decreases Na^+^-K^+^-ATPase activity. These metabolic disorders and microvascular lesions are one of the pathogeneses of DR (Wu et al., 2018). The RPE of diabetic animals shows the loss of Na^+^-K^+^-ATPase activity, and the protection of Na^+^-K^+^-ATPase activity is conducive to maintaining the calcium homeostasis of the retina and the normal signal transduction of the retina (MacGregor and Matschinsky, 1986; Chang et al., 2015; Wahren and Larsson, 2015). In previous study, ROS always used as an index for the model, but it could not show the result of AR induced by high glucose and drug under test. So, the content of Sorbitol and Na^+^-K^+^-ATPase activity were selected as index to determine the effect of high glucose, SBA and positive controls. The chemical consistent and protective effect of SBA were studied. The chemical composition of SBA was analysed by UPLC-MS/MS.16 compounds in SBA were obtained by UPLC-MS/MS analysis, including aloin, gallic acid, and ellagic acid, whose contents were 83.34 mg/g, 10.52 mg/g, and 1.01 mg/g, respectively. Phenolic derivatives from plants such as quercetin, kaempferol and ellagic acid are considered to be promising Aldose Reductase Inhibitors (ARIs) (Veeresham et al., 2014). It was reported that gallic acid, and ellagic acid have AR inhibitory activity, reduce sorbitol accumulation and improve the retinal morphology and function of the diabetic animal (Veeresham et al., 2014; Raghu et al., 2017; Marella et al., 2020). Many -OH groups in the structure of polyphenols such as gallic acid and ellagic acid may be the reason for the effective inhibition of AR. Gallic acid non-competitively inhibits AR by decreasing the turnover rate or catalytic activity of the enzyme (Alim et al., 2017). AR activity in the lens of diabetic rats was decreased by *Aloe* gel (Haritha et al., 2014). As expected, the results of the determination of the main components of SBA support its AR inhibitory activity.

Sorbitol level assay showed that 6.25–50 *μ*g/ml SBA inhibited sorbitol level in cells. After that, SBA of 100 *μ*g/ml and below was determined safe for cell viability assay. In view of the limitations of cell experiment *in vitro*, the pharmacokinetic literature of major compounds was also included as important references when setting the dose of SBA. The concentration of aloin in rodent serum after oral administration to be up to 59.07 ng/ml and the absolute bioavailability was about 5.7% by human colon adenocarcinoma cell line (Caco-2) *in vitro* intestinal absorption assay and oral plasma assay in rats (Park et al., 2008; Park et al., 2009; Niu et al., 2020), while the concentrations of gallic acid in human serum after oral administration to be up to 0.31–0.36 *μ*g/ml, and the absolute bioavailability was about 42.9% (Siranoush et al., 2001; Manach et al., 2005). According to the literature parameters and the measured values contained in SBA, the 59.07 ng/ml of aloin was calculated to apply 0.71 *μ*g/ml of SBA extract, and 0.36 *μ*g/ml of gallic acid was calculated to apply 34.22 *μ*g/ml of SBA extract, both of which were lower than 50 *μ*g/ml. Therefore, the concentrations of SBA were determined to be 6.25, 12.5 and 25 *μ*g/ml for ARPE-19 cells experiment.

In order to explore and compare the effect of SBA on AR in ARPE-19, positive controls group were designed. As the first publicly available AR inhibitor, Epalrestat has been widely used in the treatment of diabetic complications such as diabetic nephropathy and DR. Difrarel which made of *β*-carotene and bilberry [*Vaccinium myrtillus* Linn. (Ericaceae)] extract is a prescription drug for DR, *β*-carotene in it is a kind of carotenoid which belongs to AR potent inhibitor hence (Hsiao et al., 2021). Therefore, Epalrestat and Difrarel were selected as positive controls. Each 400 mg of Difrarel contains 100 mg of bilberry extract and 5 mg *β*-carotene. According to the previous report, the dose of Difrarel was designed as 25 *μ*g/ml (Mibury et al., 2007; Gong et al., 2017), and the dose of Epalrestat was designed as 0.1 *μ*M (Rajendran et al., 2014).

In our study, SBA reducing AR protein expression and inhibiting the polyol pathway. Down regulation of AR protein reduced oxidative imbalance and ROS levels, and reduced the output of sorbitol. The accumulation of sorbitol in the cells was reduced, cell edema and injury decreased, which was conducive to the recovery of Na^+^-K^+^-ATPase activity. Our results indicate that different dosages of SBA or positive controls can regulate the downstream products by inhibiting the level of AR protein and protect ARPE-19 from the metabolic disorder and oxidative unbalance caused by high glucose, which is conducive to maintaining the function of the RPE cell layer.

In response to stress, autophagy mediates the degradation and recovery of cellular components, cleans up damaged organelles and genes, and reconstructs the modules needed to maintain cell homeostasis (Boya et al., 2016). In addition, it is also considered to be the mechanism of programmed cell death, accompanied by apoptosis and necrosis. mTOR is a regulator of cell metabolism and one of the mediation targets of mTOR dependent autophagy. It forms signal transmission complexes mTORC1 and mTORC2 by binding with multiple co proteins. The ULK 1/2 is an autophagy initiation regulator regulated by the phosphorylation of upstream AMPK and mTORC1. In mammalian autophagy regulation, mTORC1 prevents AMPK from phosphorylating ULK, inhibits ULK1 activity and inhibits autophagy through the Ser758 site of phosphorylated ULK (Kim et al., 2011). On the other hand, AMPK increases its activity and initiates autophagy by directly phosphorylating the Ser555 site of ULK 1/2 (Aryal et al., 2014). We observed that the pAMPK (Thr172)/AMPK in ARPE-19 flipped twice with the prolongation of glucose induction time (1–72 h). After the significant increase of AR catalytic products (high glucose induction for 48 h), the expression of pAMPK and pULK1 increased, and the expression of pmTOR was inhibited, autophagy flow increased and autophagy was activated. After treatment with SBA, theprotein results were reversed. The positive controls Epalrestat and Difrarel also showed the same results. These results suggest that SBA or positive controls may regulate autophagy through AMPK/mTOR/ULK1 pathway.

All stages of the autophagy process are regulated by the Atg protein family. Beclin-1, also known as Atg6, plays a central role in the activation process of autophagy program. Beclin-1 can form Beclin-1-vps34-vps15 complex with class III phosphatidylinositol 3-kinase vps-34 and form phosphatidylinositol 3-phosphate (PI3P) with Atg14L, which are thought to be essential for early stages of autophagosomal formation. Bcl2 interacts with Beclin-1 through BH3 domain and destroys Beclin-1/hvps34 complexes, which is considered to be the reason for inhibiting Beclin-1 induced autophagy (Pattingre et al., 2005). In ARPE-19 of high glucose induced, autophagosomes were significantly increased, autophagy was up-regulated with excessive production of ROS, MMP was down-regulated, and Bax/Bcl2 ratio were up-regulated, which is a landmark event in the early-stage of apoptosis. Our data suggest that different dosages of SBA or positive controls reversed the results of autophagy related proteins and prevented MMP reduction and the early-stage of apoptosis. We found that SBA inhibited the appearance of autophagosomes under transmission electron microscopy.

There are still some limitations of this study. DR is a chronic disease caused by long-term exposure to hyperglycaemia. This study only established the *in vitro* model of ARPE-19 exposed to high glucose for 48 h. The high glucose exposure time should be extended to evaluate the effect of long-term high glucose exposure on AR activity and autophagy. In addition, in order to observe the effect of osmotic control conditions on the experiment, mannitol control group should be established. Therefore, in the subsequent study to evaluate the effect of SBA on RPE under longer high glucose exposure time, mannitol isotonic control group should be considered to exclude the possible damage caused by glucose osmotic pressure.

## Conclusion

In short, the intervention of SBA inhibited AR activity, inhibited autophagy through AMPK/mTOR/ULK1 and reduce the occurrence of RPE damage. This may be one of the potential mechanisms for SBA to alleviate vision loss in DR patients.

## Data Availability

The datasets presented in this study can be found in online repositories. The names of the repository/repositories and accession number(s) can be found in the article/Supplementary Material.

## Abbreviations

AR, aldose reductase; ARPE-19, acute retinal pegment epitheliitis-19 cell; Atg, autophagy-related protein; Bax, Bcl2 associated X; Bcl, B-cell lymphoma; BRB, blood-retina barrier; DCFH-DA, 2,7-dichlorodihydrofluorescein-diacetate; DR, diabetic retinopathy; mTOR, mammalian target of rapamycin; NADPH, nicotinamide adenine dinucleotide phosphate; ROS, reactive oxygen species; RPE, retinal pigment epithelium; ULK, Unc-51-like autophagy activating kinase; UPLC-MS, ultra performance liquid chromatography.
