# Supplementary material for: Shabyar Ameliorates High Glucose Induced Retinal Pigment Epithelium Injury Through Suppressing Aldose Reductase and AMPK/mTOR/ULK1 Autophagy Pathway
Source: Front Pharmacol. 2022 May 10;13:852945. doi: 10.3389/fphar.2022.852945 (PMC9127207; doi:10.3389/fphar.2022.852945)
Supplement: Supplementary file 1 [file DataSheet1.docx]

Supplementary Material

List of supplementary materials

| No. | Content | Figure/Table |
| --- | --- | --- |
| 1 | Chromatographic conditions for UPLC | Table S1 |
| 2 | Chromatographic conditions for content determination | Table S2 |
| 3 | The content of three main components in SBA | Table S3 |
| 4 | The preparation method of SBA | Fig. S1 |
| 5 | TIC diagram of methanol extract of SBA | Fig, S2 |

1. Chromatographic conditions for UPLC

| Table S2 Chromatographic conditions for UPLC | |
| --- | --- |
| Time (min) | Mobile phase ratio |
| 0 | C:4%, D:96% |
| 10 | C:18%, D:82% |
| 50 | C:24%, D:76% |
| 55 | C:50%, D:50% |

1. Chromatographic Conditions

| Table S3 Chromatographic Conditions | | | |
| --- | --- | --- | --- |
| Compound | Time (min) | Mobile phase ratio | Detection wavelength |
| Aloin | 0 | A:40%, D:60% | 355nm |
|  | 40 | A:40%, D:60% |  |
| Ellagic acid  Gallic acid | 0 | C:4%, D:96% | Ellagic acid: 254nm;  Gallic acid :273nm. |
|  | 10 | C:18%, D:82% |  |
|  | 55 | C:24%, D:76% |  |
|  | 55 | C:50%, D:50% |  |

The composition of the mobile phase is A. Equate = “methanol”, C. Equate = “acetonitrile”, D. Equate = “0.3% HCOOH-H2O”. 35 °C column temperature, with an injection volume of 10 *μ*L and a flow rate of 1.0 mL/min.

1. The content of three main components in SBA

| Table S4 The content of three main components in SBA (mg/g) | |  |
| --- | --- | --- |
| Compound | mean | SEM |
| Aloin | 83.84 | 0.04 |
| Gallic acid | 10.52 | 0.07 |
| Ellagic acid | 1.01 | 0.03 |

1. The preparation method of SBA


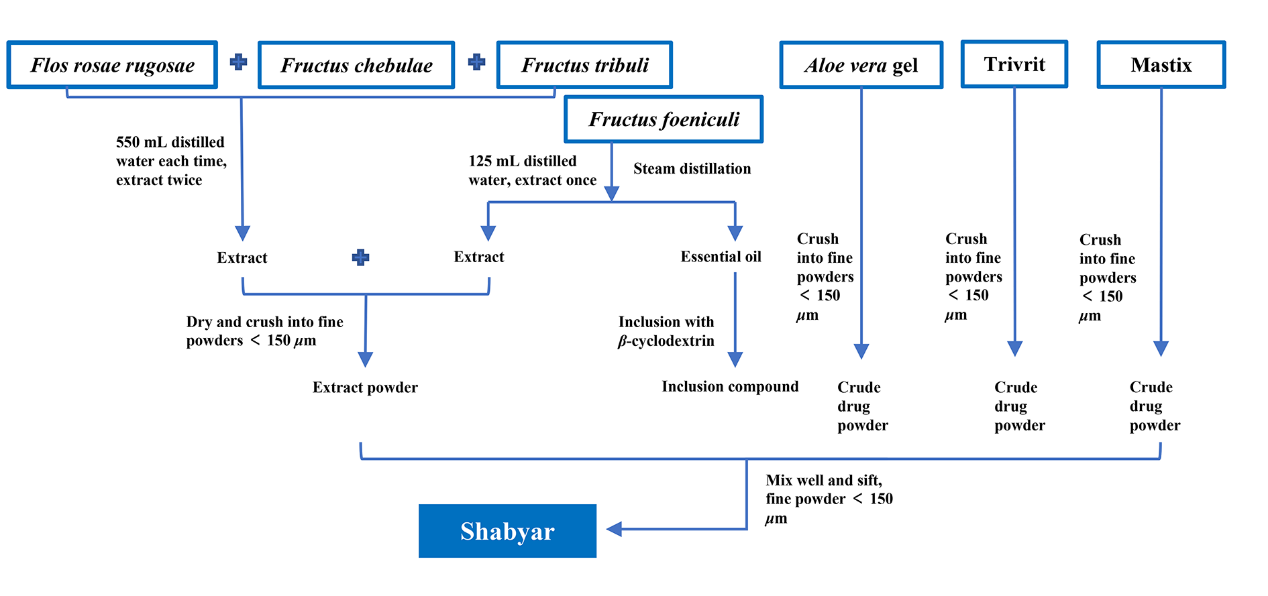


Figure S1 The preparation method of SBA

1. TIC diagram of methanol extract of SBA


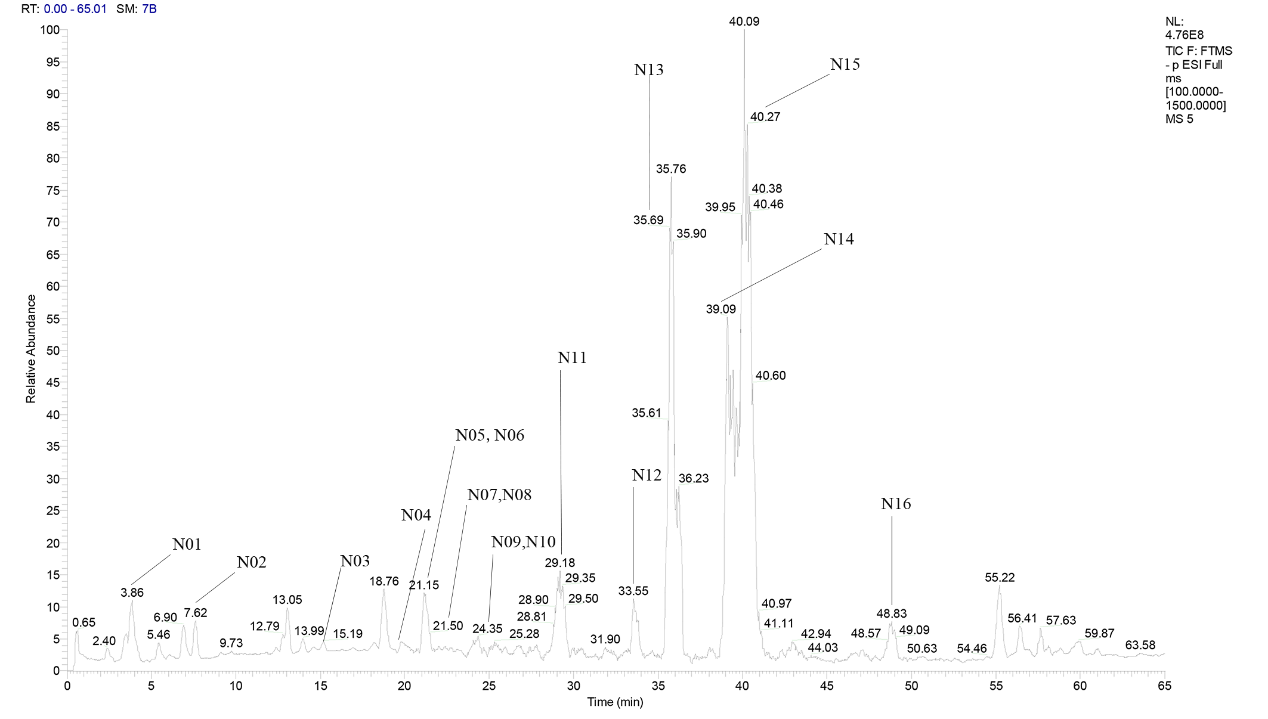


Figure S2 Total ions chromatograph diagram of methanol extract of SBA

Sixteen main chemical components in SBA were determined by UPLC-Q/TOF-MS analysis.
